# Prostate Cancer Imaging Beyond PSMA: Applications of GRPR, AR, and Amino Acid Tracers

**DOI:** 10.3390/diagnostics15212737

**Published:** 2025-10-28

**Authors:** Farzana Z. Ali

**Affiliations:** Department of Molecular and Medical Pharmacology, Division of Nuclear Medicine, University of California Los Angeles, Los Angeles, CA 90095, USA; fzali@mednet.ucla.edu

**Keywords:** prostate cancer, theranostics, GRPR, bombesin, androgen receptor, fluciclovine, molecular imaging, PET/CT, radionuclide therapy, PSMA-negative

## Abstract

Prostate-specific membrane antigen (PSMA) targeting agents have been the cornerstone of advanced prostate cancer (PCa) management in theranostics due to their high sensitivity for detecting and treating metastatic disease. However, approximately one-third of metastatic castration-resistant PCa (mCRPC) lesions may exhibit low or absent PSMA expression due to tumor heterogeneity, prior androgen deprivation therapy, or loss of androgen receptor expression, subsequently altering their response to PSMA-targeted therapy. The molecular and biological mechanisms underlying PSMA downregulation remain elusive but may include neuroendocrine differentiation or epithelial-to-mesenchymal transition (EMT). This review addresses this knowledge gap by examining recent preclinical and clinical evidence on novel radiotracers with the potential to provide alternative strategies beyond PSMA for imaging and treating PCa. The diagnostic performance and therapeutic potential of three emerging radiotracer classes are discussed, including gastrin-releasing peptide receptor (GRPR) ligands, androgen receptor (AR) ligands, and amino acid analogs. This article further highlights the complementary roles of these radiotracers along with their utility in specific patient populations, such as those with low prostate-specific antigen (PSA), biochemical recurrence (BCR), or confirmed PSMA-negative disease. For instance, GRPR-targeted radiotracers have achieved sensitivity of up to 88% and specificity of up to 90% for detecting primary tumors in PCa. The radiolabeled androgen agonist, fluorine-18 (^18^F)-fluoro-5α-dihydrotestosterone (FDHT), has demonstrated 98% true-positive rate in predicting lesions on positron emission tomography (PET) scans of mCRPC patients. On the other hand, the synthetic amino acid analog ^18^F-fluciclovine demonstrated a lesion detection rate of 84% for PSA levels at or above 5, and 62.5% for PSA levels ranging from 0.7 to less than 1. This review concludes with future directions on the paradigm of multi-tracer and dual-targeting strategies, which can effectively address challenges associated with PCa tumor heterogeneity and facilitate personalized approaches in theranostics.

## 1. Molecular Imaging in Prostate Cancer

### 1.1. Evolution of Molecular Imaging

The standard treatment for prostate cancer (PCa) confined to the prostate is radical prostatectomy or radiation therapy. When metastasis develops, androgen deprivation therapy (ADT) via surgical or medical castration may help reduce the level of circulating testosterone. Yet, the tumor cells can adapt to the castration-level testosterone via different mechanisms to promote their growth and survival [1]. This phenomenon contributes to the heterogeneous clinical behavior of PCa, widely ranging from indolent and hormone-responsive to highly aggressive and treatment-resistant [2,3]. Such tumor heterogeneity necessitates a diverse set of tools for proper diagnosis, staging, and assessment of tumor biology, with a thorough understanding of the underlying mechanisms [4]. Standard imaging modalities involved in PCa management include multiparametric magnetic resonance imaging (mpMRI) before biopsy of the prostate, in addition to thoracoabdominal computed tomography (CT) and bone scan for staging intermediate- or high-risk patients [2,5]. However, the search for higher accuracy [6], sensitivity and specificity [2,7,8] in PCa with biochemical recurrence (BCR) has led to the widespread use of molecular imaging, which offers superior performance to conventional imaging [4].

The evolution of molecular imaging in PCa has progressed through distinct phases, each addressing limitations of preceding approaches while capitalizing on advances in radiochemistry and molecular biology. Early investigations in the 1990s focused on non-specific metabolic tracers such as carbon-11 (^11^C)-choline and fluorine-18 (^18^F)-fluorocholine, which demonstrated limited specificity for prostate tissue and suboptimal lesion detection at low disease burden [9]. The subsequent development of bombesin (BBN) analogs targeting the G-protein coupled receptor, gastrin-releasing peptide receptor (GRPR), represented the first generation of truly receptor-targeted molecular imaging. Early compounds, such as DOTA-PEG(4)-BN(7-14) (DOTA-PESIN) [10] and aminobenzoic-acid (AMBA) [11], showed promise for GRPR-overexpressing PCa [12]. However, these peptide-based tracers, typically labeled with gallium-68 (^68^Ga, t½ = 68 min), required rapid imaging protocols within 1–2 h post-injection due to their fast pharmacokinetics and metabolic degradation [13]. In parallel, antibody-based strategies emerged with the development of humanized monoclonal antibodies, such as J591, which offers superior specificity and prolonged tumor retention [14]. Nevertheless, these antibody platforms necessitated longer circulation times (48–72 h) for optimal imaging contrast. They often utilized longer-lived radionuclides, such as copper-64 (^64^Cu, t½ = 12.7 h) or zirconium-89 (^89^Zr, t½ = 78.4 h), which created practical challenges in clinical implementation, including scheduling issues and concerns about radiation exposure [15,16]. While ^68^Ga-labeled peptides deliver a lower radiation burden due to their shorter physical half-life and rapid clearance, ^64^Cu-labeled compounds provide improved energy resolution (mean β + energy: 278 keV vs. 830 keV for ^68^Ga) but require specialized cyclotron production and extended imaging protocols [17]. This historical progression from metabolic to receptor-targeted tracers and from small molecules to antibodies has led to the diversification of imaging platforms, each tailored to specific clinical scenarios and the heterogeneous nature of advanced PCa. This evolution laid the groundwork for the emergence of prostate-specific membrane antigen (PSMA) as a premier target in the subsequent era of molecular imaging.

### 1.2. Rise of PSMA

PSMA is a type II transmembrane glycoprotein receptor with functional folate hydrolase and glutamate carboxypeptidase [18]. It is recognized in both native CYT-351 and immunoconjugate CYT-356 forms by the murine monoclonal antibody (mAb) 7E11-C5.3 [19]. Encoded by the Folate Hydrolase 1 (FOLH1) gene, PSMA expression is significantly high on the surface of PCa cells, particularly in poorly differentiated, metastatic, and castration-resistant forms of the disease [19], with higher expression linked to poorer prognosis [20]. PSMA has not only demonstrated superior specificity for prostate tissues but also a high contrast-to-noise ratio for PCa lesion detection [21,22]. As a result, PSMA-targeting agents have been extensively used for staging, restaging, and detecting metastasis or BCR in advanced PCa [23].

PSMA-targeted positron emission tomography (PET) imaging, using tracers such as ^68^Ga-PSMA-11, ^18^F-DCFPyL [24], and ^18^F-rhPSMA-7.3 [25], has demonstrated superior sensitivity and specificity compared to conventional imaging modalities such as CT and bone scintigraphy [26]. This is particularly evident in the setting of BCR, where PSMA-PET can detect and localize disease at very low prostate-specific antigen (PSA) levels, even below 2.0 ng/mL [27], a range where conventional scans are typically negative [23]. This capability of PSMA PET has led to earlier, more targeted interventions and novel risk stratification for patients with high-risk PCa [23].

The high expression profile of PSMA and its localization in the PCa cell membrane have paved the way for its widespread theranostic applications, where the same molecular target is used for both diagnostic imaging and therapeutic intervention [18,28]. The theranostic potential of PSMA was fully realized with the 2022 FDA approval of radiopharmaceutical therapy (RPT) using lutetium-177 (^177^Lu) PSMA-617, which combines a beta-emitting radionuclide with a PSMA-targeting ligand [29,30]. This decision was supported by key evidence from the VISION trial, which showed a longer overall survival (15.3 vs. 11.3 months) and imaging-based median progression-free survival (8.7 vs. 3.4 months) with ^177^Lu-PSMA-617 when added to standard care in patients with advanced PSMA-positive metastatic castration-resistant prostate cancer (mCRPC) [31]. Approximately 50–60% of mCRPC patients in this trial demonstrated a PSA decline of more than 50% [31]. More recently, an expanded FDA approval of this RPT in chemo-naïve patients came into being following the PSMAfore trial, which showed a significant improvement in radiographic progression-free survival with a favorable safety profile for ^177^Lu-PSMA-617, compared to switching to an alternative androgen receptor pathway inhibitor (ARPI) [32].

### 1.3. Limitations of PSMA

Despite remarkable advances in PSMA-targeted RPT, its clinical efficacy is still limited for early-stage PCa and tumors with low Gleason score [33]. Moreover, a significant subset of advanced PCa cases show a lack of PSMA expression at high enough levels to be effectively imaged or treated [34]. Histopathological studies reveal that up to 15–30% of metastatic lesions exhibit low or absent PSMA expression, with the proportion increasing in patients who have undergone multiple lines of therapy [31,35,36]. More importantly, PSMA expression involves significant intra-patient heterogeneity, with discordant expression patterns across different metastatic sites and molecular subtypes of mCRPC [37].

A previous study using an autopsy cohort of mCRPC patients found no detectable PSMA in 25%, heterogeneous PSMA expression across metastatic lesions in 44% and at least one PSMA-negative site in 63% of cases [37]. Approximately 30–40% of mCRPC patients may show no response to ^177^Lu-PSMA therapy for various reasons, including tumor biology, disease extent, and prior treatments that may contribute to heterogeneous or low PSMA expression through an aggressive trans-differentiation process [38,39]. These patients with therapy resistance usually possess visceral metastasis and lose adenocarcinoma features [39,40]. This variability in PSMA expression has a significant influence on patient selection and therapeutic efficacy of ^177^Lu-PSMA, depending on the absorbed doses in individual tumor lesions [41,42].

The PSMA-negative cohort, with distinct transcriptional profiles, represents a major diagnostic and therapeutic challenge in the management of advanced PCa [37]. Relying mainly on a negative PSMA scan may result in poor decision-making, which could include misclassifying patients, withholding or changing therapy, and undertreating high-risk populations. There remains an unmet need for the strategic expansion of therapeutic options beyond PSMA for advanced PCa. Therefore, unlike prior reviews that broadly surveyed all PCa imaging agents, this work is distinct in its exclusive focus on PSMA-negative and treatment-resistant disease phenotypes, for which alternative PET tracers are critically needed. By concentrating on cohorts with low or absent PSMA expression and those who progress despite androgen receptor pathway inhibition, this review explores potential radiotracers capable of effectively imaging and characterizing these challenging cases.

## 2. Molecular and Biological Drivers of PSMA Downregulation

The precise molecular and biological mechanisms behind the loss of PSMA expression remain to be explored. However, this is postulated to be a complex phenomenon linked to epigenetic alterations, which may include functional adaptation by the tumor to support growth and survival under therapeutic pressure [37,43]. Nonetheless, insights into the molecular mechanisms underlying this downregulation of PSMA are essential for developing rational, alternative targeting strategies for PCa.

### 2.1. Neuroendocrine Differentiation

A highly aggressive and lethal subtype of mCRPC is neuroendocrine prostate cancer (NEPC), which frequently arises after prolonged treatment with ADT or androgen receptor (AR)-targeted therapies [34,44]. This phenomenon occurs in up to 20% of advanced PCa patients who may also have resistance to hormonal treatments [44]. This process exemplifies lineage plasticity, where the tumor cells undergo a dramatic shift in their cellular identity in response to selective pressure [43]. This differentiation is characterized by a significant downregulation or loss of standard prostate-specific markers, including AR and PSMA [45]. Such suppression of PSMA expression in NEPC is likely to alter the efficacy of PSMA-targeted imaging for detecting these lesions, leading to false-negative results and limited therapeutic options with PSMA-directed RPT [46].

Following the downregulation of PSMA, these tumors often upregulate alternative cell-surface proteins, such as the somatostatin receptor 2 (SSTR2) [46]. The elevated expression of SSTR2 provides a new avenue for molecular imaging and targeted therapy [34]. The 3TMPO cohort study has examined the clinical relevance of ^68^Ga-DOTATATE PET/CT imaging in PSMA-negative mCRPC patients. It revealed that SSTR2-positive lesions are associated with poorer prognosis and shorter overall survival, thus identifying a high-risk subgroup amenable to targeted management [47,48,49]. Additionally, emerging theranostic approaches utilizing alpha-particle-labeled somatostatin analogs, such as actinium-225 (^225^Ac)-DOTATATE, have shown encouraging preclinical efficacy in neuroendocrine PCa models, paving the way for novel treatment paradigms in patients with refractory PSMA-negative disease [50]. This growing body of evidence highlights the potential of somatostatin receptor targeting as a complementary or alternative therapy option, which can provide both prognostic insights and individualized therapeutic opportunities for this aggressive tumor subtype.

### 2.2. Epithelial-to-Mesenchymal Transition

A key driver of PCa metastasis involves epithelial-to-mesenchymal transition (EMT) [51]. It is also implicated in drug resistance and poor prognosis in PCa [51]. EMT is a complex cellular programming that allows polarized epithelial cells to acquire a migratory and invasive mesenchymal phenotype. During EMT, cells lose epithelial characteristics, including cell–cell adhesion molecules, such as E-cadherin, and gain mesenchymal markers, enabling them to disseminate from the primary tumor [51].

PSMA binding occurs on epithelial cells [52], and its expression increases from benign epithelial tissue (<70%) to high-grade prostatic intraepithelial neoplasia (>75%) to malignant cells (80%) [53]. PSMA knockdown can inhibit the invasive capacity of PCa cells, indicating its functional role in disease progression [18]. While PSMA is typically overexpressed in PCa cells, the loss of PSMA expression in advanced stages can actually be a biological marker of a more aggressive, mesenchymal, and treatment-resistant phenotype [54]. Therefore, any tumor’s evolution away from a PSMA-positive phenotype should not exclude concerns about its progression towards a more lethal, invasive state [18]. In these instances, the downregulation of PSMA is likely related to the adaptive, pro-metastatic programming of the tumor [37,43]. This molecular shift provides a compelling rationale for exploring radiotracers that target the metabolic and signaling pathways that are upregulated in these aggressive, treatment-refractory cells.

### 2.3. Epigenetic and Transcriptional Mechanisms

In addition to cellular differentiation and transition processes, PSMA expression is also controlled by epigenetic and transcriptional mechanisms. One of the key transcription factors in PCa development is the nuclear receptor, AR, whose activity can significantly impact PCa risk [55,56]. AR regulates the proliferation, differentiation and survival of prostate epithelial cells through transcription of the AR gene [1]. The major circulating androgen, testosterone, and its derivative 5α-dihydrotestosterone (DHT) bind to AR, forming AR-androgen complexes that can bind to androgen-responsive elements of the tumor DNA [1], and influence tumor growth in hormone-sensitive PCa [57].

ADT can paradoxically upregulate PSMA expression in the short term [58]. However, prolonged ADT can lead to transcriptional downregulation of PSMA due to adaptation by cancer cells to the androgen-deprived environment [59,60]. This may be manifested through the loss of PSMA expression over time. Other mechanisms of epigenetic deregulation leading to loss of PSMA expression may include silencing through promoter hypermethylation [60] and a loss of histone 3 lysine 27 (H3K27) acetylation [37,61]. Researchers have demonstrated that treating cells with histone deacetylase (HDAC) inhibitors can reverse this epigenetic repression and restore PSMA expression in vitro, suggesting a potential strategy to re-sensitize PSMA-negative tumors [37].

## 3. Bombesin Analogs

### 3.1. Targeting GRPR

The GRPR subtype of the BBN family exhibits a strong affinity for gastrin-releasing peptide (GRP), a mammalian counterpart of amphibian BBN [62]. GRP is present in the nervous system and peripheral tissues, such as the gastrointestinal tract [63]. BBN analogs targeting the GRPR is present at very low levels in the normal prostate gland, but overexpressed in a wide variety of human cancers, including treatment-naïve and recurrent PCa [63,64]. GRPR is expressed in approximately 75–100% of PCas, including up to 63–100% of primary PCA, 86% metastatic lymph nodes and 53% bone metastasis [65]. Its expression in patients with low or no PSMA uptake makes it an excellent complementary target for patients with PSMA-negative disease [62]. The use of GRPR-targeting agents, particularly BBN antagonists, has gained momentum due to their superior in vivo pharmacokinetics and reduced off-target adverse effects compared to the earlier agonist versions [66].

GRPR antagonist, BAY86-7548 (^68^Ga-RM2) has demonstrated a detection rate of approximately 72% in a prospective study consisting of patients with BCR and negative findings on conventional imaging [67]. Furthermore, GRPR-targeted radiotracers have achieved sensitivity up to 88% and specificity up to 90% for detecting primary tumors in PCa [68]. These radiotracers can detect sites of PCa even at PSA levels below 0.5ng/dL [68]. ^68^Ga-labeled GRPR antagonist SB3 has demonstrated 88% sensitivity in identifying tumor lesions in biopsy-confirmed PCa patients who were therapy-naïve and scheduled for prostatectomy [69]. It has shown expression in greater than 85% of lymph node metastasis and greater than 50% of bone metastasis in PCa cases [70].

### 3.2. Clinical Development and Diagnostic Performance

Several BBN analogs have been developed for clinical use, including ^68^Ga-RM2 [71], ^68^Ga-NeoBOMB1 [72], and ^64^Cu-sarcophagine-bombesin (SAR-BBN) [73]. These agents have demonstrated high detection rates for intraprostatic and metastatic lesions [74]. A prospective trial of ^68^Ga-RM2 PET in patients with intermediate- and high-risk primary PCa demonstrated a detection rate of 93% for histologically confirmed intraprostatic lesions [75]. This radiotracer outperformed mpMRI in both sensitivity (98% vs. 77%) and accuracy (89% vs. 77%) [75].

GRPR has been recognized in majority of bone metastases originating from androgen-independent PCa [62]. A substantial increase in GRPR expression has been reported in the initial phases of malignant development, like prostatic intraepithelial neoplasia [62]. It is also highly expressed in prostate tissues that have undergone neoplastic transformation. However, it is present in only a small number of hyperplastic prostates and at very low levels in glandular and stromal tissue [62]. As a result, this tracer can aid in differentiating prostate hyperplasia or benign findings from prostate neoplasia [62,76]. The high expression of GRPR at earlier stages of PCa makes it particularly useful for initial staging [75,77]. GRPR can be an important indicator of initial molecular changes in PCa development and GRPR scintigraphy can provide a cost-effective option for early tumor detection. High RM2 uptake remains valuable for restaging, as well [63,78].

^68^Ga-RM2 is especially helpful for patients with low or no PSMA expression [63,78]. A patient with a PSMA-negative scan could still harbor a tumor that tests positive for GRPR. Such tumors might be identified using a BBN analog, potentially expanding options for diagnosis and treatment. While PSMA and GRPR tracers often show high concordance in detecting lesions, studies also reveal significant discordance [75]. A pilot study on patients with biochemically recurrent PCa showed that abdominal periaortic lymph nodes were more easily visualized with ^68^Ga-RM2 due to no interference from accumulated radioactivity in the small bowel, which showed significant physiologic uptake in ^68^Ga-PSMA [79]. Conventional imaging was noncontributory in these patients [79]. Besides, low physiological ^68^Ga-RM2 uptake in the liver facilitates the detection of hepatic lesions, making it a preferred tracer over PSMA for those with hepatic metastasis [63,80].

The variability in tracer expression within a patient directly mirrors the PCa tumor’s complex and diverse molecular profile, as different metastatic sites within the same patient may express different biomarkers. Moreover, there can be variability within each tumor lesion in patients. In those with mCRPC, intraindividual comparison of ^68^Ga-PSMA and ^68^Ga-RM2 PET scans showed a very low likelihood of simultaneous uptake in both tracers for any lesion, which relates to fundamental differences in tumor biology [78]. These two tracers should not be viewed as redundant or interchangeable; instead, they should be utilized for complementary insights [67,81]. Similarly, a multi-tracer diagnostic approach can help avoid under-staging or misclassifying patients based on a single imaging modality such as PSMA PET.

### 3.3. Theranostic Potential and Future Directions

The BBN analogs offer alternative options for not only diagnostic imaging but also therapeutic purposes in the early as well as advanced stages of PCa [63,78,82]. These agents can be used for RPT by conjugating the GRPR-targeting ligand to a therapeutic radionuclide, such as ^177^Lu or copper-67 (^67^Cu) [83]. In preclinical studies, high-dose ^177^Lu-RM2 was able to induce complete remission in six out of ten PC-3 xenograft mice [84,85]. ^177^Lu-RM2 has also demonstrated feasibility and safety with high uptake in tumor and rapid clearance from normal organs in mCRPC patients with inadequate PSMA expression or lower PSMA accumulation following prior ^177^Lu-PSMA-617 treatment [86]. These findings could have biological significance and establish a molecular foundation for potential clinical applications, such as radiotherapy with radiolabeled BBN-like peptide analogs, and chemotherapy using cytotoxic BBN analogs [62].

Patients who have previously experienced xerostomia as a dose-limiting event from PSMA-targeted RPT will particularly benefit from using ^177^Lu-RM2 if they have high expression of GRPR, since GRPR-targeted RPT has no effect on lacrimal or salivary glands [63]. More importantly, GRPR-targeted therapy can be considered in early stages of PCa, given its high expression, when PSMA often shows low expression. Additionally, similar biodistribution of PSMA and RM2 in advanced PCa suggests that alternating cycles between these two tracers for targeted RPT could reduce toxicity from each drug [63].

## 4. AR Ligands

### 4.1. AR as Imaging Target

AR is a key molecular driver of PCa growth and progression. AR gene changes involving amplification, activating mutations, increased signaling, and splice variants such as AR-V7 are major contributors to the progression of PCa, especially in the development of castration resistance [87]. These mechanisms can further sustain AR signaling despite ADT. AR-directed pathway is responsible for PSMA downregulation in response to androgen treatment [88]. Consequently, antiandrogens have been shown to upregulate PSMA expression [88]. On the other hand, PSMA knockdown can lead to activation of AR signaling [18]. Conventional markers such as PSA can serve as surrogate indicators of AR activity [89]. Yet, AR-targeted PET tracers offer a non-invasive method to directly visualize and quantify AR expression in tumors throughout the body, providing functional information on the underlying molecular biology of the disease [90].

### 4.2. AR Targeted Functional Imaging

The primary ligand of AR is the potent androgen, DHT. Its synthetic analog, 16beta-fluoro-5alpha-dihydrotestosterone (FDHT), was developed for noninvasive imaging of AR expression [91,92,93]. Loss of AR is associated with castration-resistant PCa progression to neuroendocrine type PCa, influencing response to AR-targeted therapies [94]. Among other androgen ligands evaluated in nonhuman primates, FDHT showed better and more selective uptake with greater affinity for the blood steroid carrier protein, sex hormone-binding globulin (SHBG) [57,95]. Based on those findings, this tracer was subsequently selected for human studies [57]. FDHT has shown 63% sensitivity and 86% accuracy for lesion detection in patients with advanced PCa, with positive correlation with higher PSA levels indicating greater tumor burden [89]. ^18^F-FDHT PET can provide valuable information while monitoring alterations in AR expression during PCa treatment. This can help recognize treatment-resistant behavior early in the course, providing information that can facilitate alternative strategies for halting cancer progression [96]. ^18^F-FDHT has been established as a tracer suitable for advanced PCa prognostication instead of initial staging [89,97]. ^18^F-FDHT uptake is inversely correlated to overall survival, with higher uptakes corresponding to shorter overall survival [98].

The correlation between ^18^F-FDHT PET and AR expression is much stronger than the correlation between ^68^Ga-PSMA standardized uptake values (SUVs) and PSMA expression [96]. As an androgen-repressed gene, PSMA expression is low in a hormone-sensitive state with high AR activity [88]. In patients who have progressed on ARPIs and present with a negative PSMA scan, an AR-targeted tracer such as ^18^F-FDHT could be used to determine if the tumor has regained AR signaling, a common resistance mechanism. This information on underlying molecular mechanism could guide the selection of a second ARPI or indicate the need for a different therapeutic approach.

### 4.3. Phenotyping and Clinical Integration

The rapid tumor uptake and metabolism related to ^18^F-FDHT may compromise tumor detection in regions near the prostate [57]. Thus, complementary information should be obtained using another tracer, such as PSMA. The dual tracer approach with FDHT and PSMA can help evaluate imaging phenotype heterogeneity, and aid in disease stratification and subsequent selection of patients who might benefit from androgen receptor-signaling inhibitor (ARSi) drugs [99]. Thereby, molecular subtyping informed by these tracers can facilitate rational treatment selection and move the field toward a more personalized, biology-driven treatment paradigm, ensuring that therapy is selected based on the specific molecular profile of the patient’s tumor at a given time point.

## 5. Fluciclovine as Complementary Imaging

### 5.1. Mechanism of Action and Clinical Performance

The synthetic L-leucine analog, anti-1-amino-3-fluorocyclobutane-1-carboxylic acid (FACBC) labeled with ^18^F-fluorine, also known as ^18^F-fluciclovine, was approved by the FDA in 2016 for the detection of recurrent PCa [100,101,102]. It is transported to cells by sodium-independent L-type amino acid transporter 1 (LAT1, or Solute Carrier Family 7 Member 5 or SLC7A5) and sodium-dependent alanine-serine-cysteine transporter 2 (ASCT2 or SLC1A5) [103]. These transporters are highly overexpressed in PCa compared to benign tissue [104,105].

While ^18^F-fluciclovine represents a valuable clinical tool for PCa imaging, it is important to distinguish its mechanism of action from true receptor-targeted molecular imaging agents. Unlike tracers such as PSMA ligands or BBN analogs that bind to specific cell surface receptors, fluciclovine functions as a metabolic tracer that exploits the upregulated amino acid transport pathways characteristic of malignant cells [106]. The corresponding tracer, FACBC characterizes the increased amino acid transport and metabolism associated with aggressive tumors [107]. Similarly, choline-based tracers including ^11^C-choline and ^18^F-fluorocholine operate through metabolic mechanisms, targeting the increased phospholipid synthesis associated with cellular proliferation rather than specific receptor binding [108]. Both fluciclovine and choline tracers provide functional information about tumor metabolism and cellular activity, which complements but differs conceptually from the molecular specificity offered by receptor-targeted agents [109].

Radiolabeling with ^18^F allows fluciclovine to have a longer half-life and a slower rate of renal clearance [102]. Its favorable pharmacokinetics and low urinary excretion with superior pelvic imaging (compared to PSMA) makes it particularly valuable for detecting local recurrence in the prostate bed [110,111]. In addition, it does not require an on-site cyclotron for production, which can facilitate its wider availability and use [100]. Conversely, choline tracers offer the advantage of earlier availability and established clinical protocols, though they demonstrate lower sensitivity for small-volume disease compared to both PSMA and fluciclovine approaches [109]. This metabolic targeting approach positions both tracers as complementary diagnostic tools, particularly useful when receptor-targeted imaging yields equivocal results or when assessing the hypermetabolic characteristics that distinguish malignant from benign lesions.

In preclinical studies, ^18^F-fluciclovine has shown higher target-to-background ratio as well as higher ratios of PCa to inflammation or benign prostate hyperplasia, when compared to FDG [110]. It has also demonstrated high sensitivity compared to conventional imaging for earlier detection of PCa recurrence [112]. It has particular advantages for local recurrence detection in the prostate/bed [113]. Besides, the high specificity of ^18^F-fluciclovine PET compared to conventional imaging even at low PSA levels makes it an ideal tracer for the localization of extra-prostatic disease [114]. Its capability to detect true positive local and extra-prostatic lesions increases with increasing PSA [114].

The FALCON trial demonstrated good tolerance of this tracer among 104 patients with first episode of BCR after curative-intent primary therapy [115]. The trial reported detection rates of 56%, which increased with increasing PSA [115]. The LOCATE trial showed 57% detection rate with fluciclovine PET in patients with suspected recurrence following curative intent treatment [116]. This tracer altered management plans in 59% of patients [116]. The EMPIRE-1 trial provided level 1 evidence that salvage radiotherapy planning guided by fluciclovine PET resulted in superior 3-year failure-free survival (75.5% vs. 63.0%), compared to conventional imaging [117].

### 5.2. Synergistic Use with PSMA PET

A prospective study of 50 patients with PCa BCR after radical prostatectomy showed higher detection rates (56%) with ^68^Ga-PSMA-11 PET, whereas ^18^F-fluciclovine recognized unique lesions in only 26% of cases [27]. A systematic review further reported higher detection rate of recurrence by PSMA when compared to ^18^F-fluciclovine PET (80% vs. 62%) for a PSA level of 1.0–1.9 ng/mL [102]. Although PSMA-targeted PET is generally considered more sensitive for PCa detection, ^18^F-fluciclovine can offer a unique and complementary role [27]. Their different mechanism of uptake provides an opportunity for synergistic use of PSMA’s receptor binding and fluciclovine’s metabolic targeting. ^18^F-fluciclovine could be particularly useful when PSMA results are equivocal or suspected to be false positive [118]. The non-specificity of PSMA may contribute to its expression in a benign lesion, which may not have the hyper-metabolic characteristic of a malignancy that would lead to ^18^F-fluciclovine uptake. For instance, PSMA PET can produce false-positive results in vertebral hemangiomas [118]. This was highlighted in a recent case report on a patient with a PSMA-positive lesion in a vertebral body, which was suspected to be metastasis, and was shown to be fluciclovine-negative [118]. The addition of fluciclovine helped avoid unnecessary and invasive biopsy. Therefore, these two tracers can provide distinct yet complementary information that can improve diagnostic accuracy, reduce patient anxiety, and prevent unnecessary procedures, thereby solidifying their role as an integral part of a potential refined, dual-tracer diagnostic workflow.

## 6. Comparative Analysis and Clinical Integration

### 6.1. Performance Across Tracers

A direct comparison of the aforementioned radiotracers reveals distinct performance profiles that can contribute to optimal clinical management. While PSMA PET demonstrates superior diagnostic accuracy for bone metastases, fluciclovine may provide added benefit for detecting local recurrence in the setting of low bladder activity [102,113]. Additionally, GRPR imaging can be more useful for the characterization of primary tumor in early PCa, compared to PSMA [119]. Table 1 provides a side-by-side comparison of these radiotracers in terms of their diagnostic and therapeutic applications.

### 6.2. Clinical Decision Algorithms

**Figure 1:**
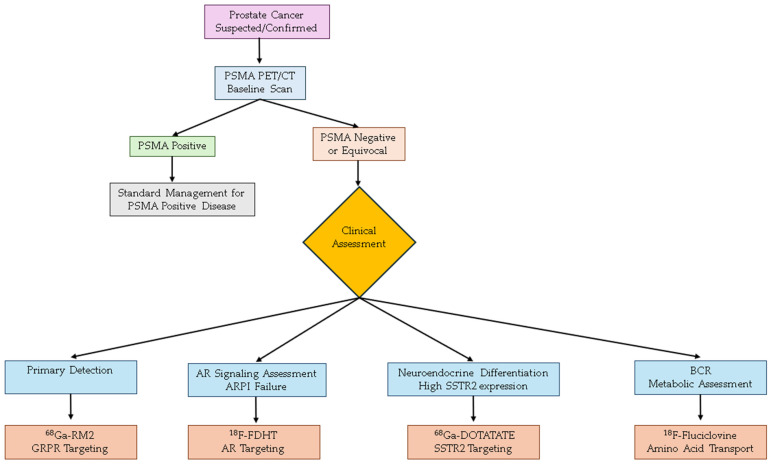
Clinical decision algorithm for selecting alternative PET tracers in prostate cancer imaging beyond PSMA. The flowchart provides guidance for tracer selection based on initial PSMA PET/CT results and subsequent clinical considerations including tumor biology, treatment history, and specific diagnostic objectives. PSMA: Prostate-Specific Membrane Antigen; PET: Positron Emission Tomography; CT: Computed Tomography; GRPR: Gastrin-Releasing Peptide Receptor; AR: Androgen Receptor; ARPI: Androgen Receptor Pathway Inhibitor; SSTR2: Somatostatin Receptor Subtype 2; BCR: Biochemical Recurrence.

Evidence-based algorithms for tracer selection (as shown in Figure 1) can be created using accumulated clinical data to improve diagnostic and treatment approaches. For the initial assessment of high-risk PCa, PSMA PET is increasingly becoming the preferred imaging modality over conventional imaging. GRPR imaging can be considered for tumors that are PSMA-negative or when conventional imaging results are discordant. PSA kinetics should be used to guide selection in BCR. Rapidly rising PSA levels with short PSA doubling times suggest aggressive tumor with potential metastatic disease, that are best evaluated with PSMA PET. On the other hand, indolent recurrence with low or slowly rising PSA levels may benefit from fluciclovine’s sensitivity for local disease. For mCRPC treatment planning, molecular imaging phenotyping may be useful for rational therapeutic selection as well as monitoring during and after treatment. PSMA-negative findings that are FDHT-positive may reflect continued AR-axis sensitivity and help with treatment selection. Lesions that show negative results on all of these tracers may need different approaches, such as chemotherapy or immunotherapy.

## 7. Theranostic Applications

PSMA tracers have the distinct benefit of the widely used ^177^Lu-PSMA-617 therapy, not available for many other alternative tracers. However, GRPR-positive lesions may also benefit from investigational BBN-based radioligand. ^177^Lu-RM2 for GRPR-targeted therapy has already completed phase I evaluation, establishing its safety and preliminary efficacy in mCRPC [86]. Future integration of alpha-particle therapy, such as ^225^Ac-DOTA-AR-BBN, can offer therapeutic advantages for micro-metastatic disease and radioresistant phenotypes [120].

## 8. Emerging Tracers and Future Perspectives

### 8.1. Novel Targets Under Investigation

The pipeline of PCa radiotracers continues to expand with novel targets addressing specific biological features. Prostate stem cell antigen (PSCA) represents a promising target with its expression on the majority of PCa. It has shown higher intensity in bone metastasis, as compared to lymph nodes or liver metastasis [121]. Carbonic anhydrase IX (CAIX), regulated by hypoxia inducible factor 1α, is upregulated under hypoxic conditions, which enables imaging of aggressive phenotypes with tumor hypoxia [122]. Additional targets, such as integrin αvβ3 [123], chemokine receptor CXCR4 [124,125], and neurotensin receptors [126] are in various stages of development.

### 8.2. Emergence of Dual-Targeting Agents

The molecular diversity of advanced PCa calls for comprehensive profiling strategies. This variability may evolve over time, and longitudinal imaging may reveal phenotypic changes driven by therapeutic pressure. A natural progression of the multi-tracer approach is the development of dual-targeting agents, which can offer more precise disease characterization. These advanced radiotracers are engineered to simultaneously bind to two distinct receptors, such as PSMA and SSTR2, within a single molecule [34]. The primary rationale behind the creation of such agents is to overcome the problem of tumor heterogeneity by ensuring a robust imaging signal even if one target is downregulated (e.g., PSMA repression) [34]. Early preclinical studies have shown that these agents can effectively bind to both PSMA-positive and SSTR2-positive tumors, opening avenues for practices where a single, comprehensive scan can provide a complete molecular profile of the heterogeneous disease [34]. This highlights the enormous potential of precision theranostics, where a single diagnostic test can guide the choice of a highly personalized and effective treatment plan.

Recent advances in PET imaging technologies, particularly the advent of long axial field-of-view (LAFOV) PET systems, have enabled the practical implementation of dual- and triple-tracer protocols that can accommodate intra- and inter-patient heterogeneity in PCa [127]. These multiparametric imaging approaches provide complementary biological insights by simultaneously assessing different molecular targets, improving lesion detection sensitivity and characterization beyond what is achievable with single-tracer studies. The superior sensitivity and extended coverage of LAFOV PET facilitate dose reduction for individual tracers while maintaining high image quality, thus mitigating cumulative radiation exposure and enhancing patient safety. Moreover, optimized imaging workflows enabled by these advanced systems reduce total scan time and improve clinical throughput, while increasing the feasibility of routine multiparametric protocols in clinical practice. The dosimetry advantages, combined with the enriched biological information obtained from multiparametric PET, are especially valuable in the context of the heterogeneous molecular landscape of advanced PCa, where different tumor clones may express variable levels of PSMA, GRPR, and other targets.

## 9. Conclusions and Future Directions

The trajectory of nuclear medicine in PCa has transitioned from non-specific imaging modalities to highly targeted, PSMA-based theranostic approaches. While PSMA PET imaging has transformed disease detection and management in PCa, its heterogeneous and treatment-resistant phenotypes call for the exploration of complementary strategies. Moreover, a single-target paradigm is insufficient to capture the full biological complexity of PCa. Emerging radiotracers targeting the GRPR, AR, and amino acid transporters offer a multifaceted diagnostic framework that reflects the tumor heterogeneity and evolutionary dynamics inherent in PCa. These agents are not ancillary but integral to the identification and characterization of aggressive, PSMA-negative or neuroendocrine-differentiated disease subtypes.

Multicenter trials should be designed for prospective validation of the clinical utility and cost-effectiveness of these tracers, with a key focus on standardizing imaging protocols and interpretation frameworks. More importantly, a multimodal approach that combines imaging results with genomic, proteomic, and liquid biopsy biomarkers can facilitate comprehensive patient stratification, where valuable insights on tumor biology can be gained through machine learning analysis.

However, in translating multiparametric and dual-tracer PET protocols from research to routine clinical practice, several practical challenges must be considered. Radionuclide availability remains a significant constraint, particularly for less common isotopes such as ^64^Cu and ^225^Ac, which require centralized cyclotron or specialized production facilities and complex supply chains [128,129]. The adoption of LAFOV PET systems, while offering unparalleled sensitivity and coverage for simultaneous tracer acquisition, is tempered by high capital costs and infrastructure requirements, potentially limiting accessibility in lower-resource settings. Furthermore, regulatory approval pathways for novel dual- and triple-tracer combinations involve rigorous safety and efficacy evaluations, and reimbursement policies vary widely across healthcare systems, creating financial and logistical hurdles for clinical implementation. Cost-effectiveness analyses comparing multiparametric PET strategies against the current PSMA PET standard are urgently needed to justify their broader adoption and to inform health-economic decision-making. Above all, ongoing collaboration among academic institutions, industry stakeholders, and regulatory agencies will be essential to streamlining the clinical translation of many of these investigative tracers for optimal patient care.

**Table 1:** Overview of Radiotracers, Key Clinical Trial Data, and Evidence Levels in Prostate Cancer.

Tracer	Primary Target	Diagnostic Role	Therapeutic Agents	Representative Trial (Design)	Approx. Sample Size (Analyzed)	Evidence Level (Phase/Design)
^68^Ga-PSMA	PSMA	High-sensitivity staging, restaging, and patient selection for RPT	^177^Lu-PSMA-617, ^225^Ac-PSMA-617	VISION (randomized, prospective, open-label) [31]	831 (randomized patients)	Phase III (screening/theranostic)
^68^Ga-RM2	GRPR	Imaging PSMA-negative tumors, primary PCa detection, neuroendocrine features	^177^Lu-RM2, ^67^Cu-SAR-Bombesin	Value of ^68^Ga-labeled bombesin antagonist (RM2) (prospective, exploratory) [130]	26 (biopsy-confirmed PCa)	Phase I/II (prospective)
^18^F-Fluciclovine	Amino Acid Transport (LAT1/ASCT2)	BCR detection, differentiating benign from malignant, metabolic tiebreaker	Limited	LOCATE [116]/FALCON [115] (multicenter)	Varied	Phase II/III (prospective)
^18^F-FDHT	AR	Functional imaging of AR expression, assessing resistance to ARPIs	Limited (diagnostic/prognostic only)	Systematic review of AR imaging studies [131]	266 (mCRPC patients reviewed)	Phase II (diagnostic/prognostic)

## Data Availability

The data related to the review is available from the corresponding author on reasonable request.

## Abbreviations

The following abbreviations are used in this manuscript:

Term	Definition
^177^Lu	lutetium-177
^18^F	fluorine-18
^67^Cu	copper-67
^68^Ga	gallium-68
ADT	androgen deprivation therapy
AR	androgen receptor
ARPI	androgen receptor pathway inhibitor
ARSi	androgen receptor-signaling inhibitor
BBN	bombesin
BCR	biochemical recurrence
CAIX	carbonic anhydrase IX
CT	computed tomography
DHT	dihydrotestosterone
EMT	epithelial-to-mesenchymal transition
FACBC	anti-1-amino-3-fluorocyclobutane-1-carboxylic acid
FDHT	16beta-fluoro-5alpha-dihydrotestosterone
FOLH1	folate Hydrolase 1
GRP	gastrin-releasing peptide
GRPR	gastrin-releasing peptide receptor
H3K27	histone 3 lysine 27
HDAC	histone deacetylase
mAb	monoclonal antibody
mCRPC	metastatic castration-resistant PCa
mpMRI	multiparametric magnetic resonance imaging
NEPC	neuroendocrine prostate cancer
PCa	prostate cancer
PET	positron emission tomography
PSA	prostate-specific antigen
PSCA	prostate stem cell antigen
PSMA	prostate-specific membrane antigen
RPT	radiopharmaceutical therapy
SAR-BBN	sarcophagine-bombesin
SUV	standardized uptake value
